# Decreased MHC I expression in IFN gamma mutant mice alters synaptic elimination in the spinal cord after peripheral injury

**DOI:** 10.1186/1742-2094-9-88

**Published:** 2012-05-07

**Authors:** Sheila CS Victório, Luciana P Cartarozzi, Rafaela CR Hell, Alexandre LR Oliveira

**Affiliations:** 1 Department of Anatomy, Cell Biology, Physiology and Biophysics, Institute of Biology, University of Campinas (UNICAMP), CP 6109, CEP 13083-970, Campinas, SP, Brazil

## Abstract

**Background:**

The histocompatibility complex (MHC) class I expression in the central nervous system (CNS) regulates synaptic plasticity events during development and adult life. Its upregulation may be associated with events such as axotomy, cytokine exposition and changes in neuron electrical activity. Since IFNγ is a potent inducer of the MHC I expression, the present work investigated the importance of this pro-inflammatory cytokine in the synaptic elimination process in the spinal cord, as well as the motor recovery of IFN^−/−^, following peripheral injury.

**Methods:**

The lumbar spinal cords of C57BL/6J (wild type) and IFNγ^−/−^ (mutant) mice, subjected to unilateral sciatic nerve transection, were removed and processed for immunohistochemistry and real time RT-PCR, while the sciatic nerves from animals subjected to unilateral crush, were submitted to immunohistochemistry and electron microscopy for counting of the axons. Gait recovery was monitored using the Cat Walk system. Newborn mice astrocyte primary cultures were established in order to study the astrocytic respose in the absence of the IFNγ expression.

**Results:**

IFNγ^−/−^ mutant mice showed a decreased expression of MHC I and β2-microglobulin mRNA coupled with reduced synaptophysin immunolabelling in the lesioned spinal cord segment. Following unilateral nerve transection, the Iba-1 (ionized calcium binding adaptor molecule 1) and glial fibrillary acid protein (GFAP) reactivities increased equally in both strains. *In vitro*, the astrocytes demonstrated similar GFAP levels, but the proliferation rate was higher in the wild type mice. In the crushed nerves (distal stump), neurofilaments and p75NTR immunolabeling were upregulated in the mutant mice as compared to the wild type and an improvement in locomotor recovery was observed.

**Conclusion:**

The present results show that a lack of IFNγ affects the MHC I expression and the synaptic elimination process in the spinal cord. Such changes, however, do not delay peripheral nerve regeneration after nerve injury.

## Background

Recent studies have shown the important role of the major histocompatibility complex class I (MHC I) expression in addition to its classical immune function. In the nervous system, its expression is directly involved in the synaptic plasticity of pre-synaptic terminals during development of the visual system, and after peripheral nerve injury in adult animals. The MHC I expression by neurons is variable and regionally localized where spontaneous electrical activity drives the establishment of adult pattern connections, including the development of the visual system and the adult hippocampus [1,2]. Mice deficient in functional MHC I show more intense pre-synaptic elimination on the surface of lesioned spinal motoneurons and reduced axonal regeneration after axotomy [3]. Indeed, the upregulation of MHC I by exogenous IFN beta treatment promotes increased synaptic stripping, leading to better gait recovery following sciatic nerve injury [4-7].

The glial cells have been implicated in the remodeling of the CNS after an injury process. Reactive astrocytes and microglia are involved in the detachment of synaptic terminals [8,9] and the release of cytokines such as IFNγ and TNFα [10,11], which promote inflammatory responses and the expression of MHC I by glial cells [12].

IFNγ has the capacity to autoregulate its expression and to modulate the expression of different genes, such as MHC I, in some neurons and glial cells [12-14]. Indeed, IFNγ may also affect neuronal differentiation and survival [15,16]. In this sense, the authors recently demonstrated that the absence of IFNγ in mutant mice altered the synaptic elimination process that results in neuronal degeneration, suggesting a neuroprotective role for this cytokine in normal animals [17].

Axonal regeneration is dependent on the neuronal response to injury and a microenvironment that favors successful regrowth of the nerve fibers from the proximal stump [8,18]. In an injury, the tissue trauma leads to an inflammatory response regulated by signaling molecules, including cytokines and other soluble factors, which are determinant for the success or failure of the subsequent regenerative outcome. Some cytokines are expressed during pathological conditions but are also involved during the acute or late recovery process following peripheral injury [19,20]. Notably, exogenous treatment with IFN beta leads to more efficient motor recovery in C57BL/6J animals after sciatic nerve crush, which has been correlated with the upregulation of MHC I by spinal motoneurons [4].

Recently, Joseph *et al*. [7] showed that the MHC I up-regulated in neurons leads to better locomotor recovery in mice submitted to spinal cord injury. These results reinforce the beneficial role of MHC I on neuro-repair after injury.

Due to current knowledge that IFNγ is a potent inducer of MHC I, the process of synaptic plasticity after injury of the sciatic nerve in animals unable to regulate the expression of MHC I via IFNγ expression was investigated using IFNγ^−/−^ mutant mice. It was observed that the spinal cord motoneurons displayed a reduced MHC I expression and presented a decreased loss of synapses one week after axotomy. Also, motor function recovery after nerve crush was improved in the absence of IFNγ.

## Materials and methods

### *In vivo* experiments

#### Animals

Adult male C57BL/6J (wild type, wt) and C57BL/6J IFNγ^−/−^ (mutant) mice (n = 5 each strain), six to eight weeks old, were obtained from the Multidisciplinary Centre of Biological Investigation (CEMIB/Unicamp) and housed under a 12-hour light/dark cycle with free access to food and water. The Institutional Committee for Ethics in Animal Experimentation approved the study (CEEA/IB/Unicamp, proc. 1172–1), and the experiments were carried out in accordance with the guidelines of the Brazilian College for Animal Experimentation (COBEA).

#### Surgical procedures and tissue preparation

The mice were anesthetized with a mixture of Kensol (xylasin, Köning, 10 mg/kg Avellaneda, Argentina.) and Vetaset (ketamine, Fort Dodge, Iowa USA 50 mg/kg, 1:1, 0.12 mL/25 g, i.p.) and were subjected to left sciatic nerve transection at the level of the obturator tendon. A 2 mm-long segment of the distal stump was removed to avoid regeneration. The muscle and skin layers were sutured, and the animals kept in the animal housing facility for one week.

Additional experiments were also carried out in order to investigate the regeneration of the sciatic nerve in both strains. The sciatic nerve was exposed at mid-thigh level and crushed at full pressure for 30 seconds with a pair of jewelers forceps (n°4), according to Xin *et al*. [21]. The muscle and skin layers were sutured, and the animals allowed to survive for two weeks (immunohistochemistry), or until complete motor function recovery.

All the animals were then sacrificed with an overdose of anesthetic, subjected to trans-cardiac perfusion with 0.1 M PBS (20 ml, pH 7.4) and then fixed with 10% formaldehyde in PBS for immunohistochemistry or with 2.5% glutaraldehyde and 1.0% paraformaldehyde in phosphate buffer (PB) pH 7.4 for electron microscopy. The lumbar enlargement of the spinal cord and the sciatic nerves were removed, cryoprotected (30% sucrose in PB for 12 hours) and frozen (immunohistochemistry) or embedded in resin (electron microscopy).

#### RT-PCR

The β2-microglobulin relative mRNA levels were determined in the right and left sides of the lumbar spinal cords of C57BL/6 J (wild type) and IFNγ−/− (mutant) mice after left sciatic nerve axotomy. The samples were mechanically homogenized and the total RNA extracted using Ribozol reagent according to the manufacturer’s instructions (Amresco, Solon, Ohio, USA). The RNA obtained from each sample (1 μg) was reversely transcribed using a commercial kit (AffinityScripts QPCR cDNA Synthesis Kit, Agilent Technologies, La Jolla, CA, USA) in a final reaction volume of 20 μL. Real time quantitative PCR was performed using the SYBR Green RT-PCR system on Mx3005P QPCR System (Agilent Technologies, La Jolla, CA, USA) after an initial denaturation for 10 minutes at 95°C, followed by 45 cycles of amplification (95°C for 30 seconds followed by 72°C for one minute). The reactions were carried out with 12.5 μL 2× SYBR Green PCR master mix (Agilent Technologies), 0.2 μM of each forward and reverse primer and 50 ng cDNA template, in a final reaction volume of 20 μL. Melting curve analyses were performed at the end of the PCR to verify the identity of the products. Melting curves occurred at 95°C for 60 seconds and 55°C for 30 seconds. All quantifications were normalized to the housekeeping gene GAPDH. A non-template control with non-genetic material was included to eliminate contamination or nonspecific reactions. Each sample (n = 4) was tested in triplicate and then used for the analysis of the relative transcription data using the 2^−ΔΔCT^ method (Livak and Schmittgen, 2001).The following forward (F) and reverse (R) primers were used: β2-microglobulin gene, (F):5′ATGGCTCGCTCGGTGACCCTG 3′ (R): 5′CCGGTGGGTGGCGTGAGTATACTT 3′. GAPDH gene, (F): 5′ TGCACCACCAACTGCTTA 3′ (R): 5′ GGATGCAGGGATGATGTTC 3′. The data are expressed as the lesioned/unlesioned ratio, adopting the unlesioned side of each strain as 100%.

#### Immunohistochemistry

The lumbar spinal cord and the sciatic nerve were frozen in liquid nitrogen at -40°C for cryostat sectioning (12 μm). The sections transferred to gelatine-coated slides were blocked in TBS-T with 3% BSA at room temperature for one hour. The spinal cord sections from axotomized mice were incubated overnight at 4°C in a moist chamber with a rat anti-MHC class I antibody (1:100, Peninsula, San Carlos, CA, USA), rabbit anti-synaptophysin (1:100, Dako, Glostrup, Denmark), goat anti-GFAP (1:200, Santa Cruz Biotechnology, Santa Cruz, CA, USA) and rabbit anti-Iba1 (1:1400, Wako Chemicals USA, Richmond, VA, USA) in TBS-T with 1% BSA. The sciatic nerves from animals submitted to nerve crush were immunostained with goat anti-p75NTR (1:200, Santa Cruz Biotechnology) and mouse anti-neurofilament (1:200, Chemicon, Temecula, CA, USA). After a further set of washes in TBS-T, the sections were incubated with CY3 or CY2-conjugated secondary antibodies (1:250, Jackson Immunoresearch, Bar Harbor, ME, USA) for one hour in a moist chamber at room temperature. The slides were then rinsed in TBS-T, mounted in a mix of glycerol/PBS (3:1), observed and documented under a fluorescence microscope (Eclipse TS100, Nikon, Tokyo, Japan) equipped with a digital camera (DXM1200F, Nikon, Tokyo, Japan).

For quantitative measurements, three alternate sections from the same level of the spinal cord (ipsilateral and contralateral sides of the spinal cord) from each animal (*n* = 5 for each group) were used to capture images from the ventral horn at a final magnification of ×20, always keeping all settings unchanged. Quantification was performed with the enhance contrast and density slicing feature of IMAGEJ software (version 1.33u, National Institute of Health, USA). The integrated density of pixels was measured in six representative areas around the motoneuron identified in the lateral motor nucleus from each side (lesioned and unlesioned sides). The lesioned/unlesioned ratio of the integrated density of pixels was calculated for each section and then as the mean value for each spinal cord. The data were represented as the mean ± standard error of the mean (SEM).

#### Electron microscopy

After fixation, the crushed sciatic nerve from each animal (n = 5 for each group) was osmicated, dehydrated and embedded in Durcupan ACS (Fluka, Steinheim, Switzerland). Ultrathin cross sections obtained from the distal stump (2.0 mm distally to the lesion site) were collected on formvar coated copper grids, contrasted with uranyl acetate and lead citrate, and examined under a Leo 906 transmission electron microscope operating at 60 kV. The number of myelinated fibers, degenerating fibers and non-myelinated axons were quantified manually using the counting measuring features of the image tool software (University of Texas, USA). Sampling bias was avoided by spreading the micrographs systematically over the entire cross section of the nerve, including all fascicles, according to the scheme proposed by Mayhew and Sharma [22]

#### Motor function recovery

For the gait recovery analysis, the Cat Walk system (Noldus Inc, Wageningen, The Netherlands) was used. In this method, the animal crosses a walkway with a glass floor illuminated from the long edge. Data acquisition was carried out by a high speed camera and the paw prints were automatically classified by the software. The paw prints from each animal (n = 5 for each group) were obtained before and after the sciatic crush. Post-operative data were assessed on the third, fourth and fifth days following surgical intervention and then twice a week until three weeks post lesion.

The parameters used herein to calculate the sciatic functional index were the distance between the third toe and hind limb pads (print length) and the distance between the first and fifth toes (print width). Measurements of the parameters were obtained from the right (normal) and left (experimental) paw prints and the values were calculated using the following formula described by Inserra *et al*. [23]: SFI = 118.9(ETS - NTS/NTS) - 51.2(ELP - NLP/NLP) - 7.5 (E = experimental side, N = normal side). Also, the pressure exerted by the individual paws during contact with the platform was evaluated. The data were expressed as the lesioned/unlesioned ratio for each day of training.

### In vitro experiments

#### Cell culture

Primary cultures of astrocytes were prepared from the cerebral cortices of one- to two- day-old C57BL/6J and IFNγ^−/−^ mice according to the method used by McCarthy and de Vellis [24]. The animals were obtained from CEMIB/Unicamp. Briefly, cortical hemispheres from neonatal mice were dissected out and, after removal of the meninges and blood vessels, were chopped and incubated in 0.05% trypsin (in PBS) for 10 minutes. DNAse was added to the pre-digested tissue, and the resulting cell suspension subjected to a 10 minute centrifugation (1,300 rpm) in 4% BSA in DMEN. The cell precipitate was re-suspended in DMEN supplemented with 10% fetal bovine serum (FBS, Nutricell Campinas, São Paulo, Brazil), penicillin and streptomycin (1 μl/ml, Nutricell), nerve growth factor (NGF, 0.25 μl/ml, Sigma Saint Louis, Missouri, USA), glucose (16 μl/ml, Nutricell) and insulin (1 μl/ml, Sigma) and seeded in cell culture flasks (25 cm^2^). The resulting primary astrocyte cultures were kept in an incubator at 37°C under an atmosphere of 5% CO_2_ for one week. On confluence, the cultures were trypsinized again and submitted to a 10 minute centrifugation. In sequence, the pellet was re-suspended in glial medium (GM) and seeded onto 24-well (2.5 × 10^4^ cells/well) cell culture plates (Corning/Costar Corporation, Cambridge MA, USA). These plates were placed in an incubator under the same conditions (37°C, 5% CO_2_). The GM was renewed every other day and all the experiments were performed in triplicate.

### Immunocytochemistry

One week after culturing, the astrocytes were fixed with 4% paraformaldehyde in (D)MEM, rinsed several times in PBS and incubated in TBS-T with 3% BSA at room temperature for one hour. The cultures were further incubated for two hours with the primary antibody goat anti-GFAP (1:100, Santa Cruz) and rat anti-MHC I (1:200, Santa Cruz) diluted in TBS-T with 1% BSA. After incubating with the primary antisera, the cultures were rinsed in TBS-T and incubated for 45 minutes with Cy3-conjugated secondary antisera (1:250, Jackson Immunoresearch). The preparations were then mounted in a mixture of glycerol/PBS (3:1), observed using an inverted microscope (Nikon eclipse TS100) and quantified using IMAGEJ software (version 1.33u, National Institutes of Health, USA). For the GFAP labeling analysis, the integrated density of pixels was measured at six random areas in each well (four wells in total). The average of the integrated density of pixels was calculated for each well and then for each group and compared between them. For the MHC I immunolabeling, the integrated density of pixels was measured over the entire image surface and the value divided by the total number of nuclei counted. The average labeling was normalized per 1 × 10^5^ of surface area. The data were represented as the mean ± standard error of the mean ± SEM.

#### Analysis of cell proliferation

New culture plates were used for the cell proliferation measurements. Twenty four hours after culturing on 24-well plates, three wells per group were fixed with 4% paraformaldehyde (Reagen, Colombo, Parana, Brazil) in (D)MEM, while development and growth continued in the other well. The same fixation procedure was carried out every other day up to one week after culturing.

The fixed cultures were submitted to immunocytochemistry against a proliferation marker, namely the PCNA proliferating cell nuclear antigen (PCNA). The cells were incubated with rabbit anti-PCNA antibody (1:200, Santa Cruz) for two hours and then for 45 minutes with Cy3-conjugated secondary antibody (1:250, Jackson Laboratories). Nuclei labeling was performed with 4′,6-diamidino-2-phenylindole (DAPI, Dako) for 10 minutes, the cultures rinsed in phosphate buffer (0.1 M PBS), mounted in a mixture of glycerol/PBS (3:1) and observed under an inverted microscope (Nikon eclipse TS100) connected to a Nikon camera (DXM1200F). The number of astrocytes was determined with the ImageTool software (version 3.0, UTHSCSA, USA) by counting all the DAPI stained nuclei. The mitotic rate was calculated from the ratio of PCNA/DAPI labeling in 15 randomly obtained areas, documented for each day of culture fixation. Three points (second day, fourth day and sixth day after culturing) were obtained from the mean value calculated for each group studied.

#### Statistical analysis

The data are presented as mean ± SEM, and the differences between groups were considered significant when the P-value was <0.05 (*). Statistical analysis was performed with Graphpad Prisma 4.0 software. In this sense, data were subjected (ANOVA) followed by the Bonferroni post hoc test for parametric data or the Mann–Whitney *U* test for non-parametric data.

## Results

### Changes in the MHC class I expression in the absence of IFNγ

**Figure 1:**
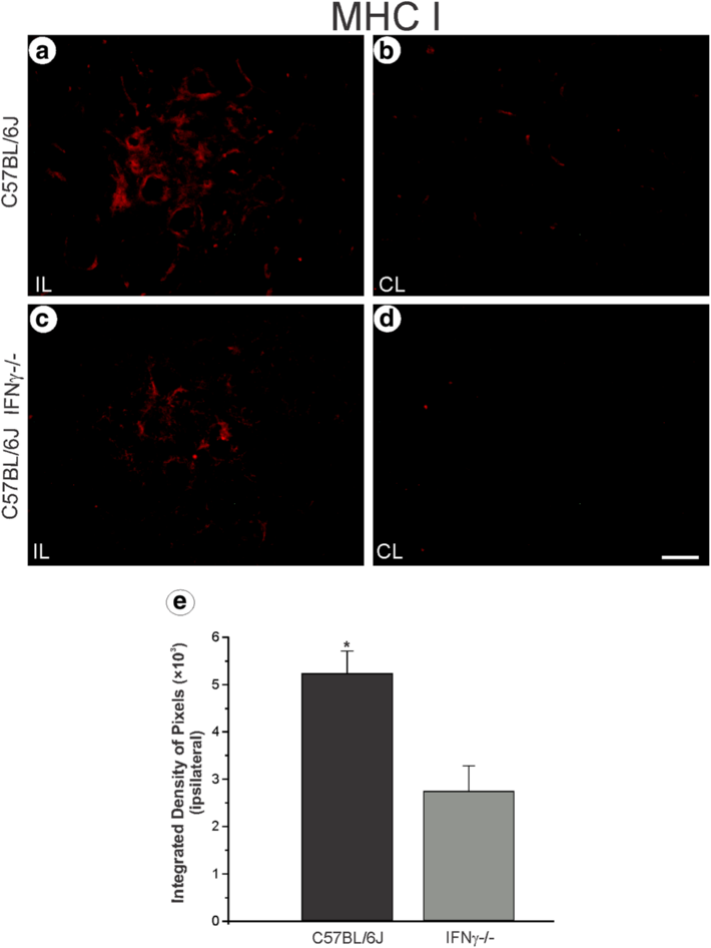
**The MHC class I expression is increased in C57BL/6J (A, B) as compared to the IFNγ−/− mice (C, D).** This fact was confirmed by the quantification of the integrated density of pixels in the neuropils adjacent to large motoneurons. (**E**; p < 0.05). Scale: 50 μm. MHC, major histocompatibility complex.

One week after axotomy a clear change in the MHC class I levels could be detected in the lesioned motoneurons from both strains. The protein expression was clearly greater on the lesioned side for both strains (Figure 1A, C compared to 1B, D). However, the mutant mice showed a lower MHC class I expression around the motoneurons as compared to the wild type (C57BL/6J, 5.21 ± 0.48; C57BL/6J IFNγ^−/−^, 2.72 ± 0.53; *P* < 0.05; Figure 1E), indicating an influence of the lack of IFNγ in the upregulation of MHC class I.

**Figure 2:**
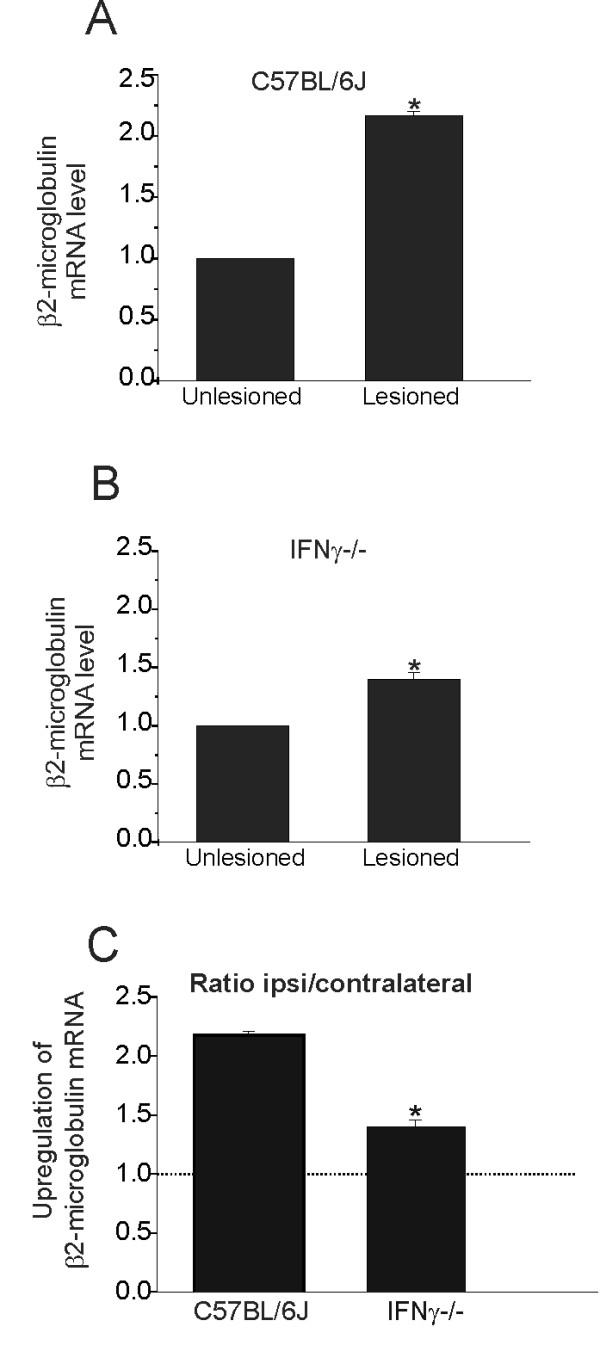
**The graph shows β2-microglobulin mRNA upregulation following unilateral sciatic nerve axotomy determined by RT-PCR (P < 0.05).** The unlesioned side was used as reference (100%). (B) β2-microglobulin mRNA level in the lumbar spinal cord of IFNγ^−/−^ mice. The graph shows a smaller β2-microglobulin mRNA upregulation following unilateral sciatic nerve axotomy determined by RT-PCR (*P* < 0.05), as compared to the wild type strain. The unlesioned side was used as reference (100%). (C) β2-microglobulin mRNA upregulation in C57BL/6J and IFNγ^−/−^ mice (ratio ipsi/contralateral). Observe that the mutant mice display a smaller upregulation of β2-microglobulin following lesion. * = *P* < 0.05.

The RT-PCR was performed to examine the transcriptions of β2-microglobulin in the absence of IFNγ. The results showed a significantly enhanced β2-microglobulin mRNA expression in the lesioned side of the wild type as compared to mutant animals (C57BL/6J, 2.17 ± 0.03; C57BL/6J IFNγ^−/−^, 1.4 ± 0.05; *P* < 0.05; Figure 2).

### Synaptic covering was affected in IFNγ^−/−^ mice but not glial reactivity

**Figure 3:**
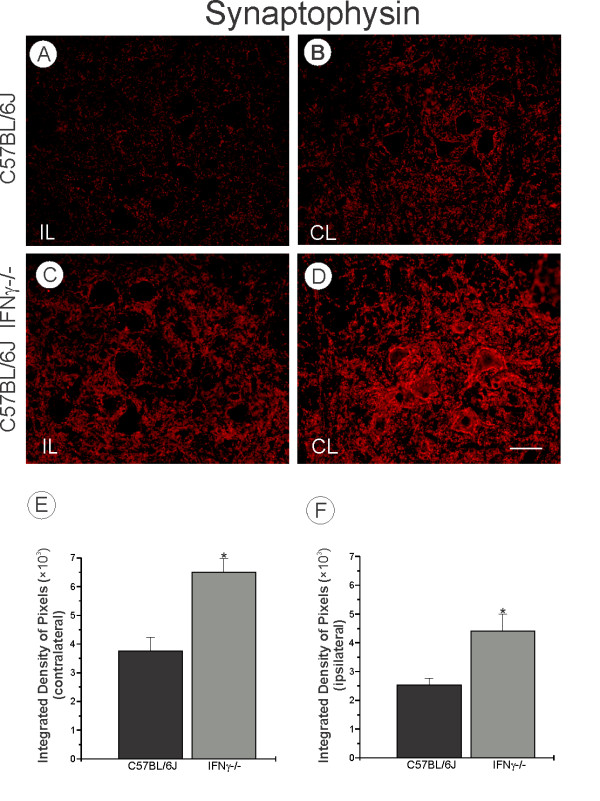
**(A, C) Ipsilateral side (IL, lesioned), showing an overall reduction of the labeling in the wild type as compared to the mutant mice.** (**B**, **D**) Contralateral side (CL, unlesioned) shows greater labeling in the mutant mice. These results were confirmed by quantification of the integrated density of pixels (**E**, **F**). Graph **G** represents the ipsi/contralateral ratio, showing no statistical differences between the C57BL/6J and IFNγ^−/−^ mice (*P* > 0.05). Scale: 50 μm.

In order to assess the changes in synaptic activity after peripheral lesion, the spinal cord sections were immunostained with synaptophysin. The labeling found in the contralateral ventral horn was compared with that on the ipsilateral side. As seen in Figures 3A and 3C, there was reduced synaptic elimination on the lesioned side of the mutants as compared to the same side of the wild type strain (C57BL/6J IFNγ^−/−^, 4.41 ± 0.6; C57BL/6J, 2.54 ± 0.23; *P* < 0.05) (Figure 3E). Even on the unlesioned side (Figures 3B and D), the mutant animals showed a higher expression of synaptophysin than the wild type (C57BL/6J IFNγ^−/−^6.51 ± 0.47; C57BL/6J, 3.77 ± 0.46; *P* < 0.05; Figure 3F). However, an analysis of the ipsi/contralateral ratio one week after axotomy indicated a similar response to injury for both strains (C57BL/6J IFNγ^−/−^, 0.71 ± 0.09; C57BL/6J, 0.71 ± 0.08; p > 0.05; Figure 3G). The results suggest that the lack of IFNγ leads to synaptic changes in the mutant animals in a way that is independent of the lesion.

**Figure 4:**
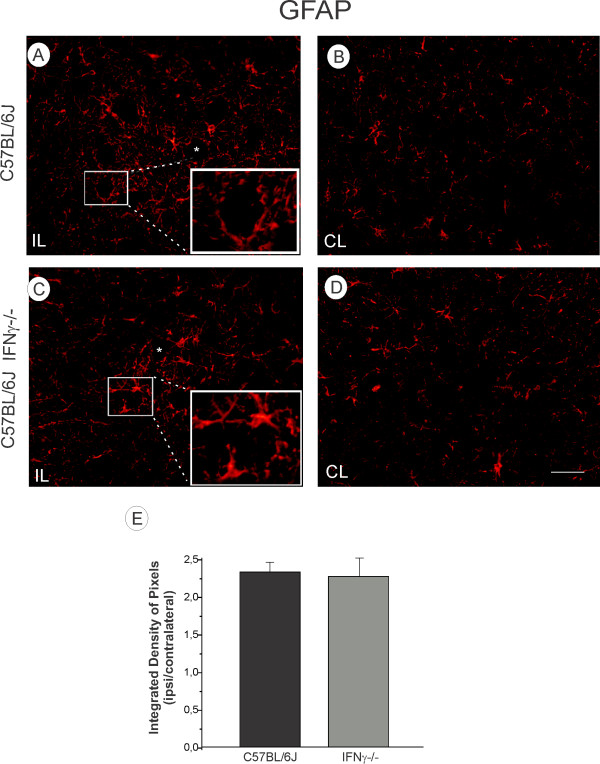
**(A, C) Ipsilateral side (IL, lesioned) of the C57BL/6J and IFNγ−/− mice, respectively.** The lesion upregulated GFAP labelling in both strains. (**B**, **D**) Contralateral side (CL, unlesioned) of the lumbar spinal cord. Observe the presence of more hypertrophied astrocytes surrounding motoneurons in the IFNγ^−/−^ mice (inset in A) as compared to C57BL/6J (inset in C). (**F**) Graph representing the ipsi/contralateral ratio (p > 0.05). Scale: 50 μm.

**Figure 5:**
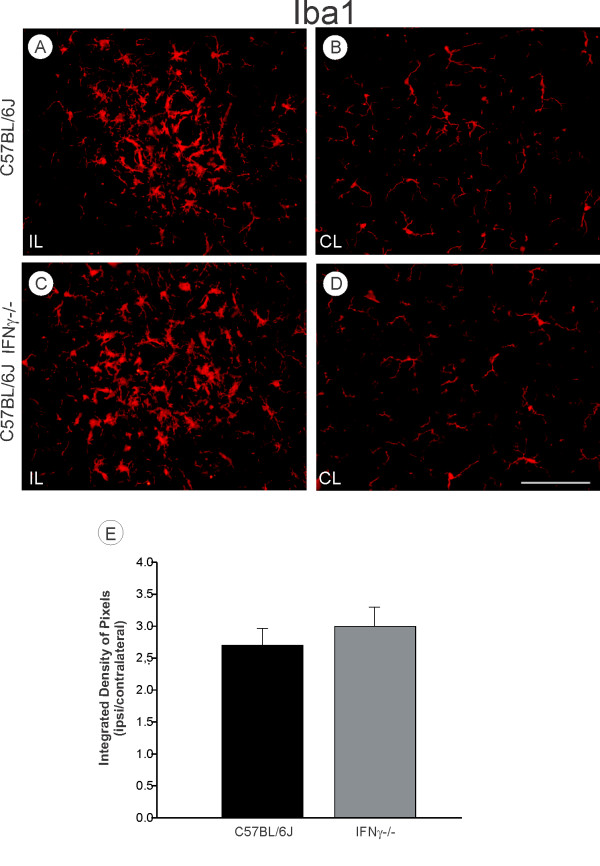
**(A, C) Ipsilateral side (IL, lesioned).** The Iba 1 expression increased, particularly on the surface of the axotomized neurons of both strains, but no differences between the experimental groups were detected (p > 0.05). (**B**, **D**) Contralateral side (CL, unlesioned) of C57BL/6J and IFNγ^−/−^ mice. Scale: 100 μm.

Immunoreactivity for GFAP was used to assess the differences in astroglial reactivity one week after axotomy. The unlesioned side showed a basal expression of GFAP (Figure 4B, D), whereas on the lesioned side, GFAP-reactivity increased in both strains (Figure 4A, C). This was evident in the micro-environment close to large motoneurons in the sciatic nerve pool. Nevertheless, the quantitative analysis showed no statistical differences between the strains (C57BL/6J, 2.33 ± 0.13; C57BL/6J IFNγ^−/−^, 2.27 ± 0.24; *P* > 0.05; Figure 4F). On the other hand, although the integrated density of pixels measurement did not reveal differences between the strains, more hypertrophied astrocytes were observed in knock out samples (Figure 4A, C). This finding, combined with the *in vitro* data described below, may indicate that the lack of IFNγ leads to an increase in hypertrophy but a decrease in hyperplasia following injury. The Iba-1 expression (a microglial marker) was negligible in the unlesioned samples of either strains (Figures 5B and D), indicating that mutant and wild type mice displayed equal basal microglial cell reactivity. An increase in Iba-1 expression could be seen on the lesioned side of both strains (Figures 5A and C), suggesting that the lesion, but not the lack of IFNγ, upregulated the microglial reactivity (C57BL/6J, 2.7 ± 0.26 e C57BL/6J IFNγ ^−/−^, 3.0 ± 0.3; *P* > 0.05; Figure 5E).

### Reactivity of the Schwann cells in the absence of IFNγ

**Figure 6:**
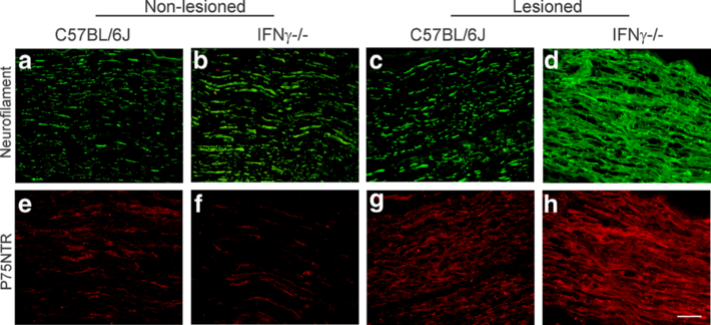
**Note that the neurofilament and p75NTR labeling are more intense in the mutant mice in comparison to the wild type.** Scale: 50 μm.

Sections of the crushed nerve (distal stump) were labeled with anti-neurofilament, an intermediate filament component of the axon cytoskeleton. The mutant mice showed an elevated expression of this axonal marker, as well as an altered organization of the fibers following lesion, as compared to the wild type (Figures 6C and D). The immunostaining of the unlesioned nerve showed low levels of p75NTR (Figures 6E and F). However, after sciatic nerve lesion, this receptor was strongly up-regulated in the mutant mice as compared to the wild type (Figures 6G and H).

### Number of degenerated and regenerated axons

**Figure 7:**
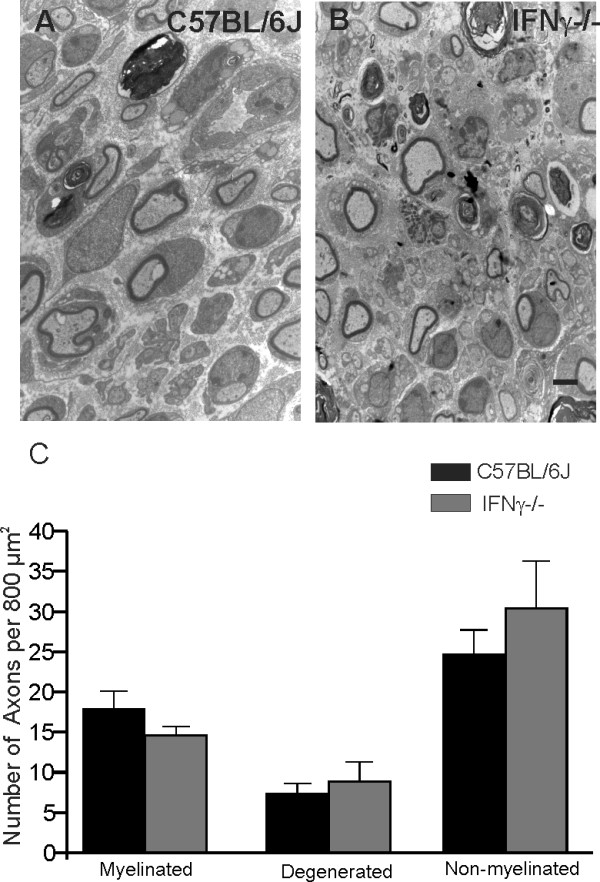
**Graph (D) shows the quantification of the nerve fibers and axons.** There was no statistical difference between the groups. Scale: 1 μm.

Two weeks after crush, non-myelinated axons, myelinated fibers and those undergoing degenerative processes were evaluated (Figures 7A and B). The results showed a higher number of non-myelinated axons in the mutant mice, although no statistical differences could be identified between the mice studied (C57BL/6J, 24.76 ± 2.94; C57BL/6J IFNγ^−/−^, 30.46 ± 5.79; *P* > 0.05; Figure 7C). Also, a small number of degenerated fibers could be seen in both strains (C57BL/6J, 7.41 ± 1.18; C57BL/6 J IFNγ^−/−^, 8.92 ± 2.34; P > 0.05; Figure 7C).

### Evaluation of motor function in IFNγ^−/−^ mice after crush

**Figure 8:**
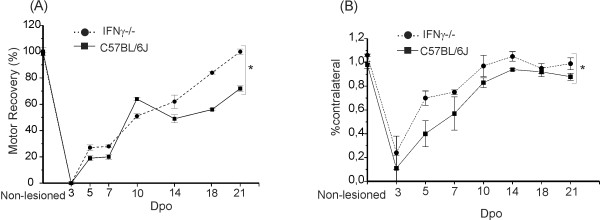
**(A) Graph of the sciatic functional index (SFI) values according to the group and time of evaluation.** The data are presented as the percentage of the preoperative values. (**B**) The intensity of the paw print is shown as a percentage of the contralateral paw during 21 days post-operative (Dpo).

The recovery of motor function was studied by the walking track test using the Cat Walk System (Noldus Inc). The sciatic function index was first calculated from the distance between the first and fifth toes (the width of the foot print) and the distance between the third toe and the hind pad (the foot print length). Before the lesion, the mutant mice showed a lower value of the sciatic function index as compared to wild type mice. After the initial loss of function (immediately after the crush lesion), motor recovery increased progressively and it was statistically faster in mutant mice (*P* = 0.0129, two way ANOVA). One week after lesion, the mutant animals showed a 28% improvement as compared to 20% in the wild type. The following week, the functional recovery was 62% in the mutant mice and 49% in the wild type, and 21 days post operative, the mutant mice showed 100% of motor function recovery, while the wild type only reached 72% (Figure 8A).

The results for toe pressure on the walkway revealed that the mutant group supported more weight on the ipsilateral paw after a shorter period of recovery in comparison with the wild type. Three weeks after surgery, the mutant mice had recovered 99% of the paw print and the wild type 88%. In this sense, a significant difference was obtained between the groups for this parameter, based on a two-way ANOVA (*P* = 0.0025), that took into account the strain type and the recovery time period (Figure 8B).

### IFNγ regulation of GFAP and MHC I in the cultures

**Figure 9:**
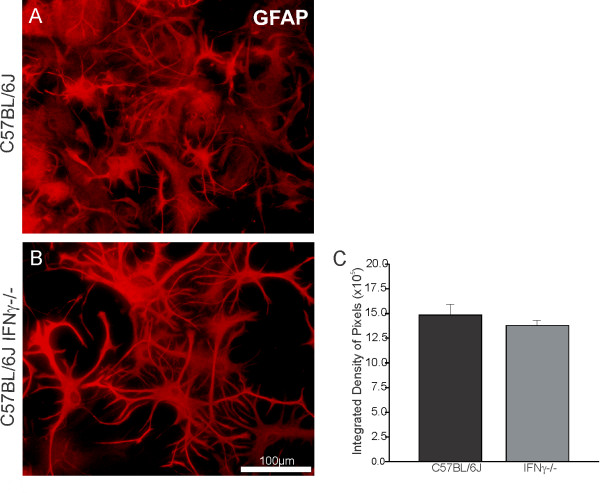
**(C) Graph demonstrating no statistical difference in GFAP immunolabelling between the experimental groups (P > 0.05).** Scale bar: 100 μm. GFAP, glial fibrillary acid protein.

The astroglial reactivity was also investigated *in vitro* in astrocyte primary cultures from mutant and wild type mice, which were isolated and maintained under the same experimental conditions. The results showed no difference in GFAP immunoreactivity between the strains (C57BL/6J, 14.76 ± 1.08; C57BL/6J IFNγ^−/−^, 13.22 ± 0.50; P > 0.05; Figure 9C), in line with what was seen *in vivo*. However, a higher proliferation rate could be observed in the cultures from the wild type as compared to the mutant-derived preparation (Figure 9A, B).

**Figure 10:**
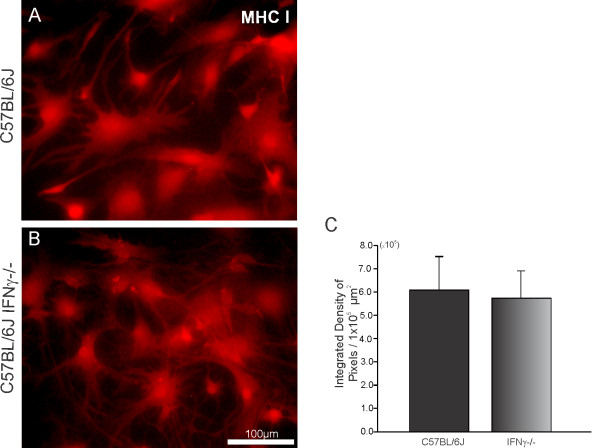
**(C) Graph demonstrating no statistical difference in MHC I immunolabeling between the experimental groups (P > 0.05).** Scale bar: 100 μm. MHC, major histocompatibility complex.

There was also no substantial difference between the groups with respect to MHC class I immunoreactivity (C57BL/6J, 6.00 ± 1.67; C57BL/6J IFNγ^−/−^, 5.62 ± 1.37; *P* > 0.05; Figure 10). Both animal groups showed the same level of MHC class I expression in culture, which was different from the *in vivo* observations.

### Absence of IFNγ affected the proliferation of astrocytes *in vitro*

**Figure 11:**
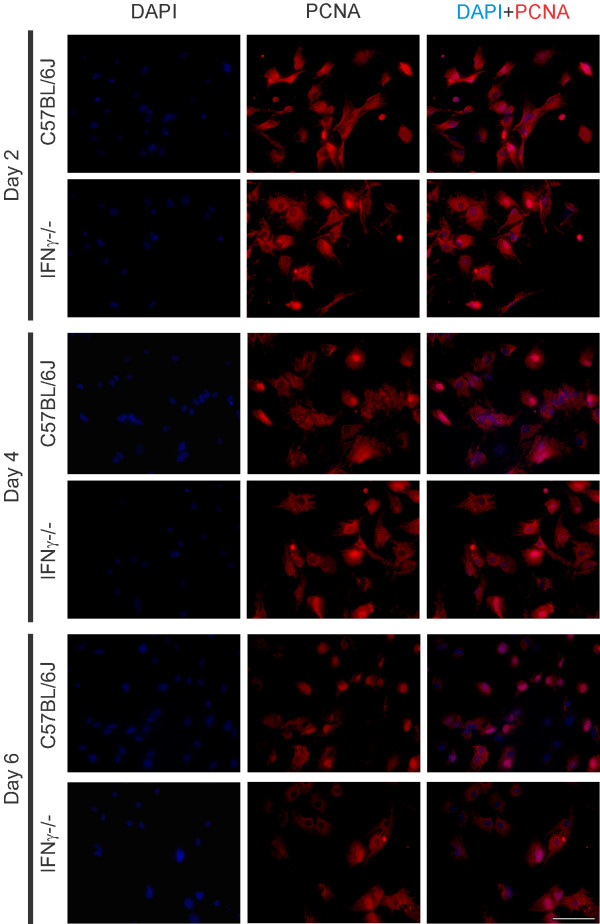
**Observe the greater amount of nuclear staining in C57BL/6J derived cultures especially at the sixth day of culturing.** DAPI, 4′,6-diamidino-2-phenylindole; PCNA, proliferating cell nuclear antigen.

**Figure 12:**
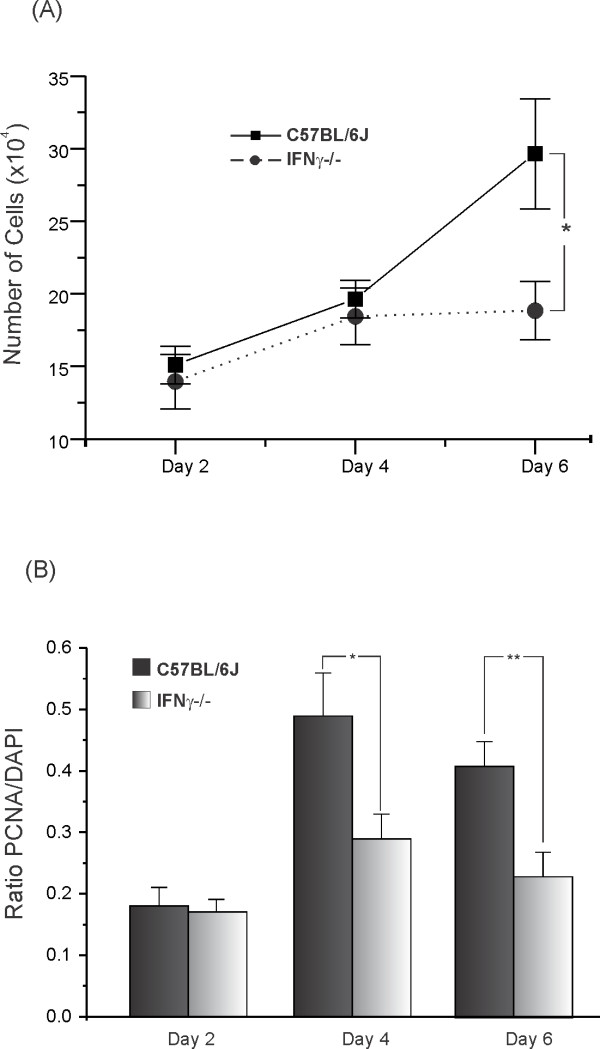
**(A) The mutant cell cultures displayed a significantly slower proliferation rate during the period analyzed. (B)** The absence of IFNγ affected the cell growth ratio as compared to the C57BL/6J cultures. (*P < 0.05; **P < 0.01). Scale: 100 μm.

To confirm whether the absence of IFNγ had any effect on astrocyte proliferation, DAPI staining of nuclear DNA was employed to analyze the number of viable astrocytes in both cultures. Figure 11 shows representative images of the cultures during the entire experiment. The images indicate a greater number of PCNA-positive cells in the wild type cultures than in the mutant cultures. After 24 hours of culture, the total number of cells was obtained every other day in order to plot a growth curve. As can be seen in Figure 12A, the absence of IFNγ had a significant effect on the astrocyte proliferation rate. After four days of culturing, there was no further increase in the number of astrocytes in the mutant derived culture, to the contrary of the control culture. The differences were significant on the sixth day, when control cells began to proliferate rapidly (C57BL/6J, 29.65 ± 3.8; C57BL/6J IFNγ^−/−^, 18.84 ± 2.0, *P* < 0.05).

The mitotic rate was analyzed by immunostaining with PCNA, and calculated from the ratio of PCNA/DAPI labeling. After 24 hours of culturing, no statistical differences were obtained between the experimental groups, but in the following days, the mitotic rate in the control cultures were statistically greater than in the mutant counterparts (Figure 12B).

## Discussion

The correlation between the MHC I expression and the success of axonal regeneration after lesion, has been widely discussed. The lack of functional MHC I induces synaptic changes in the spinal motoneurons, specifically a higher synaptic elimination of inhibitory inputs to the axotomized motoneurons. Indeed, such changes resulted in a poorer regenerative potential of the regenerating axons [3]. On the other hand, MHC I upregulation by exogenous stimulus [4,6] or even in animals with an enhanced MHC I expression [5,7], was shown to produce a more effective regenerative outcome after lesion.

MHC I mRNAs and protein have been found in normal animals in different groups of neurons, such as in spinal motoneurons [25], dorsal root ganglion neurons [14], hippocampal neurons [13,26], cortical pyramidal cells [1,2] and glial cells [27]. MHC I and β-microglobulin RNAm expressions were previously studied by Lindå *et al.*[25] in normal and axotomized motoneurons. Indeed the enhanced MHC I expression after sciatic nerve lesion was accompanied by IFNγ receptor RNAm upregulation in large motoneurons localized in the lamina IX in the spinal cord. IFNγ is a potent inducer of MHC class I and is produced and secreted by resident cells of the CNS [10]. The presence of inflammatory cytokines, such as TNF and IFNγ, has been implicated in neuronal damage and recovery [20] but the action of cytokines remains unexplored in the normal CNS. Therefore, the role of MHC I in the synaptic plasticity and axonal regeneration of animals that lack IFNγ expression was evaluated after sciatic nerve transection and crush. The results showed that the absence of IFNγ did not alter the basal levels of MHC I *in vivo* or *in vitro* (primary astrocyte cultures). However, the MHC I protein expression as well as β2 microglobulin mRNA on the lesioned side of IFNγ^−/−^ mutant mice was lower than in the wild type mice, indicating that the absence of this cytokine reduces the MHC I upregulation induced by unilateral sciatic nerve transection. Such a difference may be related to the expression of MHC class I on the surface of the neurons, which requires the correct assembly of class I heavy chain, the β2-microglobulin and the antigenic peptides. In this sense, the assembly mechanism of MHC class I subparts with the expressed peptide is not totally understood. However, the role of IFNγ activated proteasome has been pointed out as an important part of the process [28,29]. Likewise, experiments have demonstrated that TNF, IFNβ and also IFNγ increase the steady-state levels of MHC class I mRNA and also the corresponding cell surface expression [13,30,31], which might explain the lower levels of mRNA β2-microglobulin obtained in the mutant mice.

The loss of MHC I signaling led to decreased synaptic elimination in mutant mice as shown by immunohistochemistry. These results are in agreement with the findings of Huh *et al*. [2], where the lack of MHC I hampered the synaptic elimination process during development of the visual system. In addition, a quantitative ultrastructural analysis of lesioned motor pools showed a lower detachment of synaptic terminals from the surface of α-motoneurons in IFNγ^−/−^ mutant mice as compared to the wild type [17].

Following axonal lesion, a prominent retrograde reaction occurred in the neural cell bodies and activation in the surrounding glia. Recent studies suggest that reactive glial cells are involved in modulating the synaptic processes, inducing displacement of the presynaptic terminals from axotomized motoneuron bodies [9,32]. The present results revealed a greater glial reaction in both groups after unilateral sciatic nerve transection. Interestingly, mutant mice presented more prominent cells processes, indicating hypertrophy, but not hyperplasia. *In vitro*, the astrocytes appeared equally reactive in terms of MHC I and GFAP expression; however, the proliferation rate in cultures from the wild type was higher than in the mutant-derived cells. These results suggest that the lack of IFNγ affected the astrocytic response to injury, reducing cellular proliferation (but possibly increasing hypertrophy in a compensatory way) rather than increasing cell death [16]. Additionally, since there appears to be a correlation between synaptic density and astroglial activity in the spinal cord microenvironment, a smaller number of astrocytes around the axotomized neurons, as the *in vitro* data suggest, would not lead to a greater loss of synaptic contacts in the knockout group compared to the control. This is in line with the concept that a decreased loss of synapses is related to the proper activation of astrocytes.

It is important to emphasize, however, that equal *in vitro* amounts of MHC I expression in primary astrocyte cultures from both strains has to be interpreted with caution. This may be a side effect of the lack of cross talking with microglial cells, which are activated earlier then astrocytes following injury *in vivo*. The absence of such interaction, due to the cell purification procedures, reinforces the role of microglial cells in the overall response to lesion as well as in the synaptic elimination processes that follow.

The upregulation of the MHC I expression has been reported after injury, showing the importance of this molecule for the success of the regenerative outcome. The exogenous treatment with IFNβ enhanced the CNS response to injury by upregulating the MHC I [4] and improving the motor recovery of the animal treated with this cytokine [6]. Additionally, animals with enhanced neuronal MHC I expression showed faster and better locomotor recovery after spinal cord injury [7]. Our results showed that the IFNγ^−/−^ mutant mice presented an enhanced p75NTR and neurofilament expression after nerve crush, which could indicate a more efficient response of the peripheral nervous tissue of the mutant mice as compared to the wild type, positively affecting axonal re-growth. Possibly other inflammatory pathways may have been activated during Wallerian degeneration, thus compensating for the absence of IFNγ in these animals. Nevertheless, there was no significant difference in the number of axons found in the distal stumps in the two groups. The gait recovery analysis, on the other hand, showed that the overall motor recovery of the IFNγ^−/−^ mutant mice was significantly faster than that of the wild type mise for the parameters analyzed. This is an interesting result, indicating that the absence of such pro-inflammatory cytokine may be beneficial to the recovery of peripheral nervous system, although it appears to have a different role in the spinal cord microenvironment, especially on astrocytes, contributing to a decreased synaptic elimination process.

In conclusion, it was shown that the absence of IFNγ altered the MHC I expression and that such negative regulation affected the synaptic elimination process of the spinal cord. However, the modifications in the spinal microenvironment did not affect the motor function recovery, which was faster that in the wild type strain. A better understanding of the IFNγ action beyond the known immune function may provide new information regarding its importance in MHC I signaling and its consequences for the nervous system maturation and regeneration.

## Conclusions

Altogether, the present study showed that the absence of IFNγ affected the MHC I expression and altered the synaptic elimination process in the spinal cord. Although the lack of IFNγ did not alter the morphological parameters of the peripheral nerve regeneration after nerve injury, it accelerated the overall motor recovery of mutant mice.

## Abbreviations

BSA, Bovine serum albumin; CNS, Central nervous system; GFAP, Glial fibrillary acid protein; Iba-1, Ionized calcium binding adaptor molecule 1; IFNγ−/−, Nterferon gamma knock out; MHC I, Major histocompatibility complex of class I; RT-PCR, Reverse transcriptase-polymerase chain reaction; SEM, Standard error of deviation; TEM, Transmission electron microscopy; TNF, Tumor necrosis factor.

## Competing interests

The authors declare that they have no competing interests.

## Authors’ contributions

ALRO provided the study concept, design and supervision. SCSV participated in the experimental design and data acquisition. LPC and RCRH participated in acquisition of some experimental results, particularly the RT-PCR data. ALRO and SCSV provided an analysis and interpretation of the data and took part in writing the manuscript. All the authors read and approved the final manuscript.
